# Overexpression of mutant *Ptch* in rhabdomyosarcomas is associated with promoter hypomethylation and increased Gli1 and H3K4me3 occupancy

**DOI:** 10.18632/oncotarget.3272

**Published:** 2015-03-14

**Authors:** Frauke Nitzki, Ezequiel J. Tolosa, Nicole Cuvelier, Anke Frommhold, Gabriela Salinas-Riester, Steven A. Johnsen, Martin E. Fernandez-Zapico, Heidi Hahn

**Affiliations:** ^1^ Department of Human Genetics, University Medical Center, Göttingen, Germany; ^2^ Schulze Center for Novel Therapeutics, Division of Oncology Research, Mayo Clinic, Rochester, MN, USA; ^3^ Microarray and Deep-Sequencing Core Facility, University Medical Center, Göttingen, Germany; ^4^ Department of General, Visceral and Pediatric Surgery, University Medical Center Göttingen, Göttingen, Germany

**Keywords:** RMS, Ptch, Gli1, DNA hypomethylation, H3K4me3

## Abstract

Mice with heterozygous loss of the tumor suppressor *Patched1* (*Ptch*) develop rhabdomyosarcoma (RMS)-like tumors. However, *Ptch* transcripts are consistently overexpressed in these tumors. We have recently shown that the upregulated transcripts are derived from the mutated *Ptch* allele thus leading to the hypothesis that the wild-type allele is repressed during RMS development. Here we describe epigenetic changes taking place at the *Ptch* locus during RMS development. We showed a lower degree of DNA-methylation in methylation-sensitive CpG regions of the *Ptch* promoter in RMS compared to normal muscle from heterozygous *Ptch* animals. In agreement with these results, treatment of heterozygous *Ptch* mice with the DNA demethylating agent 5-aza-2-deoxycytidine (5-aza-dC) between embryonic days E9.5–E11.5 significantly accelerated RMS formation. Since *Ptch* promoter methylation occurs after/around E13.5, the window for RMS initiation during embryogenesis, these results provide additional evidence that *Ptch* promoter hypomethylation may contribute to RMS formation. We have also demonstrated increased trimethylation of histone H3 lysine 4 (H3K4me3) and preferential binding of Gli1, a known Ptch activator, to the mutant locus in RMS. Together, these findings support an alternative model for RMS formation in heterozygous *Ptch* mice including loss of methylation and concomitant occupancy by activating histone marks of mutant *Ptch*.

## INTRODUCTION

Hedgehog (Hh) proteins signal via two multitransmembrane proteins, Ptch and Smoothened (Smo). In this receptor complex, Ptch is the ligand-binding subunit and Smo is the signaling component. Upon binding of Hh to its receptor Ptch, an inhibitory effect of Ptch on Smo is released, allowing Smo to trigger a signaling cascade activating the Gli transcription factors (Gli1, 2 and 3), essential effectors for Hh-mediated cellular effects. Due to its repressive function in the Hh pathway, inactivation of Ptch results in pathological activation of Hh signaling that in turn promotes tumor development. This is characterized by increased levels of *Gli1* mRNA (reviewed in Hooper *et al.* [1]). Since the *Ptch* promoter contains Gli-binding sites, activation of the Hh pathway is also followed by *Ptch* transcription [2, 3].

Inactivating *Ptch* mutations have been identified in various tumors including basal cell carcinoma, medulloblastoma and RMS [4–6] thus supporting a role for *Ptch* as a tumor suppressor gene. However, one normal allele is frequently retained in the tumors [7–9]. Using heterozygous *Ptch* knockout mice we demonstrated that the wildtype (wt) and mutated *Ptch* alleles are differentially expressed in RMS. Interestingly, the *Ptch* transcripts consistently overexpressed in RMS are derived predominantly from the mutated allele, whereas transcript levels of the wt allele appear to be unchanged or even downregulated [10–12]. These observations led to the hypothesis that RMS formation in heterozygous *Ptch* knockout mice is associated with epigenetic mechanisms differentially repressing mutant and wt *Ptch* alleles.

Since DNA methylation is one of the best characterized epigenetic modifications contributing to the repression of genes with tumor suppressor activity in tumors, we initially examined the levels of DNA methylation at the *Ptch* promoter in RMS to compare the DNA methylation status. We identified specific changes in DNA methylation that are associated with transcriptional activation of mutant *Ptch* in tumors. In addition, we show an increased occupancy of activating histone marks between normal and tumorigenic tissue of heterozygous *Ptch* knockout mice as well as Gli1 binding to the mutant *Ptch* allele. Finally, we monitored RMS formation in heterozygous *Ptch* knockout mice that have been treated with 5-aza-dC at the RMS-susceptible stage E9.5–E11.5 during embryogenesis. Moreover we analyzed the methylation status of the *Ptch* promoter in mouse embryos at E9.5 and E13.5 (i.e. the RMS-susceptible and RMS-non-susceptible stages, respectively [12]). Altogether, our findings define a pattern of epigenetic changes associated with differential expression of the mutant and wt allele and provide foundation for future studies aimed at defining the mechanism underlying the interplay between these marks and Gli1 in RMS pathogenesis.

## RESULTS

### The mutant *Ptch* promotor is hypomethylated in RMS from Ptch heterozygous mice

**Figure 1:**
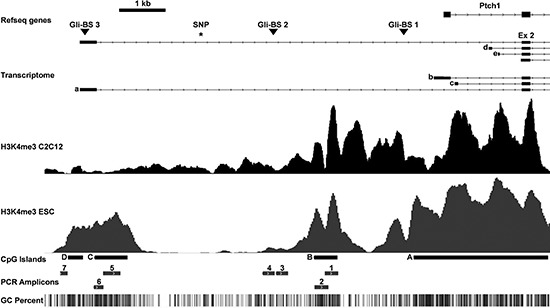
Murine *Ptch* promoter region analyzed in this study The upper part shows the upstream genomic region of *Ptch*, its transcriptome, the three Gli-binding sites (triangles) and the SNP rs29624336 (asterisk). The alternative exons 1a-1e (a–e) and exon 2 (ex 2) are indicated. H3K4me3 occupancy in differentiated C2C12 cells and murine embryonic stem cells (ESC) as downloaded from the European Nucleotide Archive and NCBI Gene Expression Omnibus are indicated below. The CpG islands (A–D), the analyzed PCR amplicons 1–7 (fragment 3 equates the methylation-sensitive DNA fragment described by Ecke *et al*. [11], all primers are shown in supplemental Table 1) and the CpG content are shown in the lower part. The largest amplicon 5 (391 bp) could only be amplified in the H3K4me3 ChIP experiment and NGS bisulfite sequencing.

**Figure 2:**
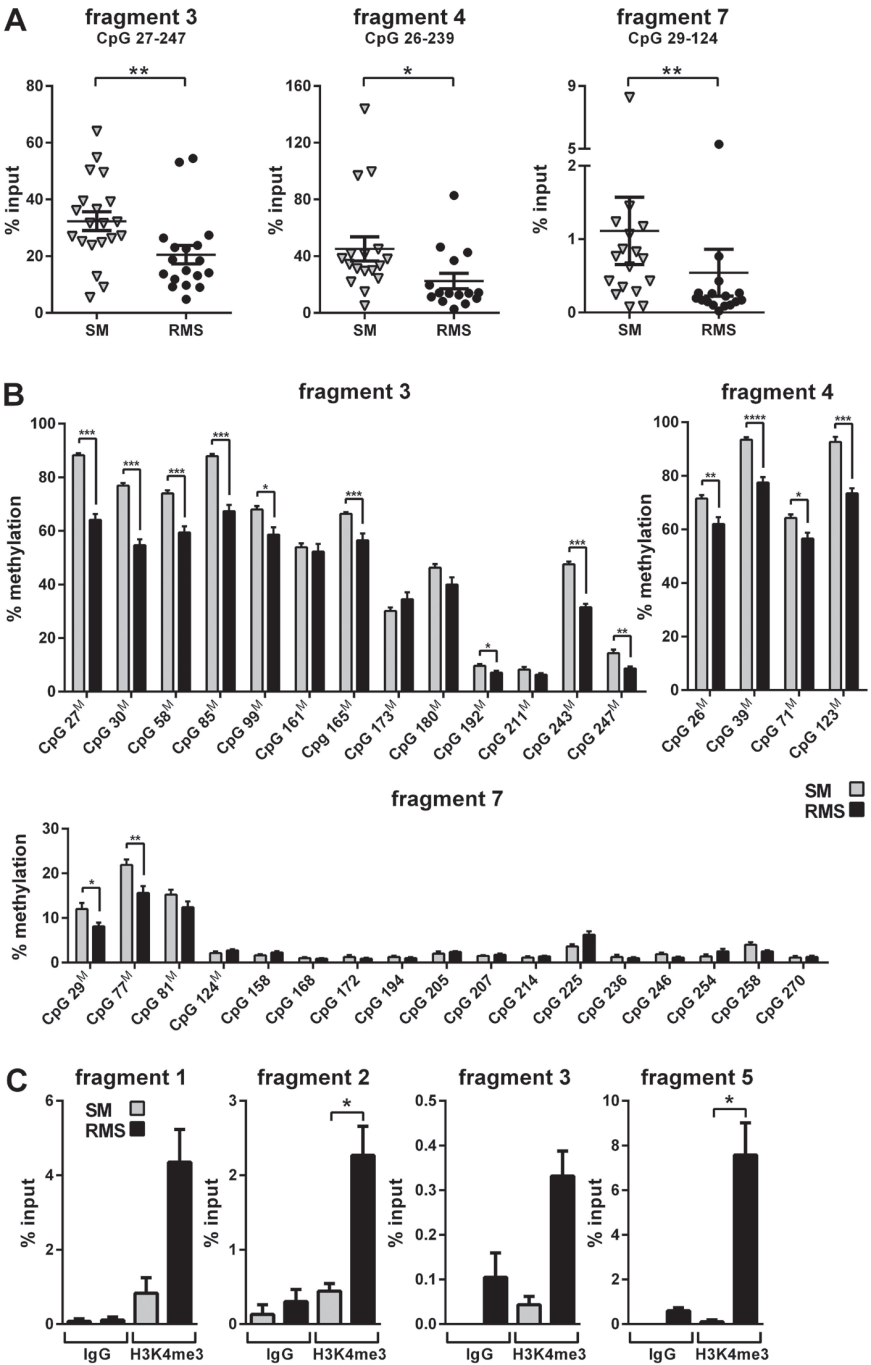
Analysis of methylation and histone occupancy of the *Ptch* promoter in RMS of heterozygous *Ptch* mice **A and B.**, Decreased methylation of the *Ptch* promoter in RMS. A, DNA derived from tissue samples from *Ptch*^+/−^ (*n* = 10) and *Ptch^neo67/+^* (*n* = 6) mice was subjected to MeDIP and analyzed by qPCR. Each triangle (SM) and circle (RMS) represents one sample measured in triplicates. Mean values (lines) and SEM are indicated for each group. The decrease in methylation in amplicons 3, 4 and 7 in RMS is statistically significant. The covered CpG dinucleotides (in comparison to bisulfite NGS, see B) are indicated for each fragment. B, Decreased methylation of several CpG dinucleotides in the *Ptch* promoter in RMS. SM (grey bars) and RMS (black bars) from 9 *Ptch*^+/−^ mice were analyzed by bisulfite NGS. Mean values and SEM from SM and RMS are given for each CpG at the indicated position within the respective fragment. CpG dinucleotides also covered in the MeDIP assay are marked with a superscript^M^. **C.**, H3K4me3 enrichment at the *Ptch* promoter in RMS. The ChIP enrichment for H3K4me3 was analyzed in chromatin from RMS (black bars) and SM (grey bars) from 1 *Ptch*^+/−^ and 3 *Ptch^neo67/+^* mice and quantified by qPCR. Mean values and SEM for the amplicons 1, 2, 3 and 5 are shown, IgG served as a negative control. The increase in H3K4me3 occupancy in fragments 2 and 5 in RMS is statistically significant. All statistically significant differences are indicated by asterisks (*P < 0.05, **P < 0.01, ***P < 0.001, ****P < 0.0001).

We recently showed that RMS of *Ptch* heterozygous mice consistently overexpress the mutant *Ptch* allele whereas the expression of the remaining wt *Ptch* allele is unchanged or even repressed [11, 12 and Supplementary Fig. S1]. Further *in silico* analysis identified a methylation-sensitive region within the murine *Ptch* promoter in RMS [11, Fig. 1] that suggests DNA methylation as potential mediator of this phenomenon. We next analyzed methylation of the *Ptch* promotor by MeDIP using RMS and SM samples from 16 *Ptch^del/+^*(*Ptch*^+/−^) [13] and *Ptch^neo67/+^* [14] mice. The total methylation content was calculated as % input. As shown in Fig. 2A, 3 of the 7 amplicons analyzed showed methylation, whereas the remaining amplicons were unmethylated (data not shown). Surprisingly, and in contrast to our previous hypothesis, the methylation content in these fragments (i.e. amplicon 3, 4 and 7 of the *Ptch* promoter) was reduced in RMS compared to SM (Fig. 2A). Thus, the percentage of methylation in SM in the amplicons 3, 4 and 7 decreased from 32% to 20% (*P* = 0.0053), from 45% to 22% (*P* = 0.0112) and from 1.1% to 0.5% (*P* = 0.0054) in RMS, respectively. Although the decrease was small, especially in fragment 7, the difference between SM and RMS was statistically significant. For positive and negative controls for the MeDIP see supplemental Fig. S2. Next, we used bisulfite next generation sequencing (NGS) to validate the above results. Due to the special requirements of bisulfite specific primers, the amplicons differed slightly from the MeDIP (as indicated in Figs. 2A and 2B). In agreement with the MeDIP results, methylation was only detected in the fragments 3, 4 and 7. Moreover we found a highly significant decrease of methylation at several CpG positions in RMS compared to SM (9/13 CpG positions in fragment 3, 4/4 in fragment 4 and 2/17 in fragment 7). As a control we analyzed two amplicons within the CpG island A (see Fig. 1, Supplemental Table 1) close to the Gli-binding site 1 and we were able to confirm the lack of methylation in this region ([11] and data not shown).

We next sought to determine whether the methylated or unmethylated amplicons in RMS were derived from the transcriptionally repressed wt *Ptch* or the transcriptionally active *Ptch* mutant allele, respectively (see also supplemental Fig. S1). Allele-specific methylation is a widespread phenomenon that also occurs tissue-specifically and can be identified by the presence of specific Single Nucleotid Polymorphisms (SNPs) [15]. We therefore searched for SNPs in the amplicons 3, 4 and 7 (see Fig. 1) that would have allowed for discrimination between the mutant and the wt *Ptch* alleles when breeding *Ptch*^+/−^ mice to another mouse strain. Unfortunately, no informative SNPs within these amplicons were present according to the Mouse Phenome Database and to our bisulfite NGS analyses. We were therefore unable to investigate allele specific methylation.

### Enhanced occupancy of the *Ptch* promoter by activating H3K4me3 marks in RMS

**Figure 3:**
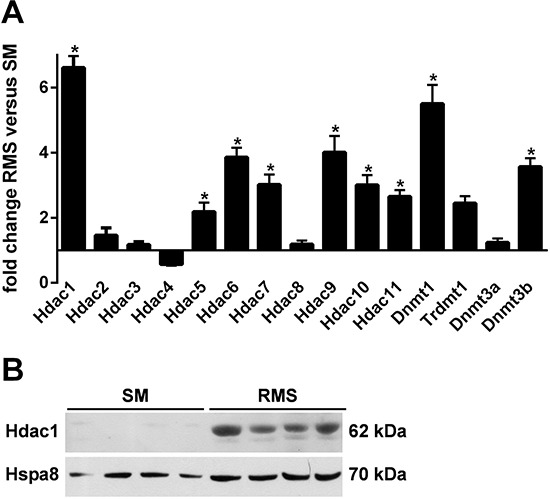
(A) Expression of *Hdacs*, *Dnmts* and *Trdmt1* in SM and RMS of heterozygous *Ptch* mice Tissue samples were isolated from 4 *Ptch^neo67/+^* mice and analyzed by qPCR. Shown is fold expression of the genes analyzed in RMS (mean ± SEM) in comparison with normal skeletal muscle (SM) that was set to 1. Statistically significant changes in gene expression levels between RMS and SM are marked with an asterisk (*P* < 0.05). **B.** increased expression of Hdac1 protein in RMS. Hdac1 was analyzed in protein lysates from RMS and SM derived from 4 *Ptch^neo67/+^* mice. Hspa8 served as loading control.

We extended the promoter analysis and investigated if changes in histone marks were accompanying the DNA methylation patterns of the wt and mutant *Ptch* alleles. Specifically, we examined the levels the trimethylation of H3K4, a hallmark of unmethylated CpG islands [16]. We immunoprecipitated H3K4me3-modified chromatin and performed qPCR (supplemental Table 1, for controls see supplemental Fig. S3). We found that the *Ptch* promoter contained 4 regions that were occupied by H3K4me3 in RMS, but not in SM derived from 4 *Ptch* heterozygous mice (Fig. 2C). Increased H3K4me3 enrichment was observed for amplicons 1, 2, 3 and 5. The difference between RMS and SM was statistically significant for amplicons 2 and 5 (both *P* = 0.0286). These data are consistent with ChIP-sequencing data for H3K4me3 in C2C12 mouse myoblast cells and murine ESC and together demonstrate an increased occupancy of H3K4me3 on the *Ptch* promoter in RMS (Fig. 1). Next, we determined what were the candidate mediators of these changes in RMS and normal SM. Interestingly, there was no significant changes between tumor and normal tissue in enzymes writing or erasing H3K4 marks including *Kmt2a* [17], *Ash1l* [18], *Cxxc1* [19], *Kdm1a* [20] and *Ezh2* [21] (supplemental Fig. S4). However, the histone deacetylases (*Hdac*) *1, 5–7* and *9–11* [22] as well as DNA methyltransferases *Dnmt1 and 3b* [23] were significantly increased in RMS compared to SM (Fig. 3A, 3B). The high levels of *Hdac1* and *Dnmt1* in RMS were previously reported to be induced by active Hh signaling [24, 25]. In contrast, the *de novo* DNA methyltransferase *Dnmt3a* did not show aberrant expression in RMS although it was reported to be regulated by Gli1 as well [25]. Together, these data show increased levels of the active histone modification H3K4me3 in the *Ptch* promotor in RMS and increased expression of enzymes involved in DNA methylation and histone deacetylation in the tumors.

### RMS show higher binding of Gli1 at the mutant *Ptch* promoter

To determine how the methylation differences contributed to the differential transcriptional activity of the *Ptch* alleles in RMS, we initially examined the binding of Gli1, a known activator of Ptch in cancer, in SM and RMS from 5 *Ptch*^+/−^ mice and consecutively amplified the fragments 1–4 and a 197 bp fragment covering the polymorphism by qPCR (Supplemental Fig. S5 and data not shown). For fragment 4 covering a Gli-binding site (Gli-BS 2, Fig. 1) increased Gli1 binding was found in RMS. A slight increase in RMS was also detected in the neighboring fragment 3, whereas no difference was detected for the other amplicons (Supplemental Fig. S5). To determine if there were differences in the binding of Gli1 between alleles we made use of the SNP rs29624336, a genetic variant allowing to differentiate between the genetic backgrounds 129Sv and Balb/c (C) and C57BL/6 (T) (supplemental Fig. S6). When crossing *Ptch*^+/−^ mice (on a Balb/c background) in which the vector used for the propagation and original targeting was 129Sv [26] to wt C57BL/6 mice, the mutant locus in the resulting Balb x B6 *Ptch*^+/−^ mice carries the C allele and the wt *Ptch* locus the T allele. Due to the location of the SNP rs29624336 in vicinity to the Gli-binding sites indicated in Fig. 1 (~1.6 kb to Gli-BS 2 and ~2.6 kb to Gli-BS-3) this SNP serves as excellent reference to determine if the level binding of Gli1 was enhanced at the mutant locus. To this end, we used 3 RMS from Balb x B6 *Ptch*^+/−^ mice. DNA was precipitated with the Gli1 antibody and after qPCR was subcloned and sequenced. Indeed, although the results did not reach statistical significance, in all samples the C allele and thus the mutant *Ptch* locus was more frequently amplified than the T allele (together 83 C and 59 T). These data show that Gli1 binding is higher at the mutant *Ptch* allele. This together with hypomethylation of the mutant *Ptch* alleles in RMS may explain the higher transcriptional activity of the mutant *Ptch* allele.

### 5-aza-dC treatment during embryogenesis accelerates RMS development in heterozygous *Ptch* mice

**Figure 4:**
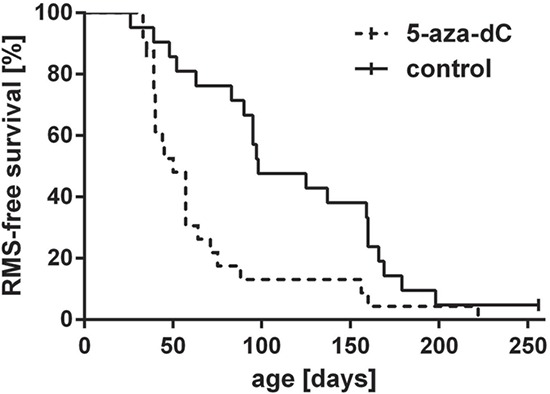
Lifetime monitoring of RMS in heterozygous *Ptch* mice after exposure to 5-aza-dC Pregnant mice were injected with 5-aza-dC at 9.5, 10.5 and 11.5 dpc and formation of palpable RMS in the *Ptch*^+/−^ offspring (dashed line) was monitored weekly until the age of 250 days. *Ptch*^+/−^ control animals (continuous line) were not exposed to 5-aza-dC. Two censored animals are marked by a bar. The difference between both groups is statistically significant (*P* = 0.0012, Gehan-Breslow-Wilcoxon test).

Using a conditional knock-out strategy in *Ptch^flox/+^* mice, we recently demonstrated that disruption of one *Ptch* allele at embryonic day E9.5 in the mouse results in RMS initiation in approximately 90% of mice. The RMS susceptibility declines to 44% when *Ptch* is disrupted at E11.5 and the window for RMS initiation is closed at E13.5 during embryogenesis [12]. These data suggest that epigenetic modifications at the *Ptch* promoter repressing the wt allele while concomitantly activating the mutant *Ptch* allele occur before day E13.5. If hypomethylation of the *Ptch* promoter indeed plays a role in RMS formation, global DNA demethylation by 5-aza-dC at the RMS-susceptible stage should either influence onset, incidence or latency time of RMS in *Ptch*^+/−^ mice. To test this hypothesis, we mated female wt mice to *Ptch*^+/−^ males and treated the pregnant animals with 5-aza-dC at 9.5, 10.5, and at 11.5 days post coitum (dpc) (i.e. at the RMS-susceptible stages). At the end of the study 92% and 95% of 5-aza-dC and untreated animals, respectively, developed at least one visible or palpable and histologically confirmed RMS (Fig. 4 and Table 1). There were no differences in morphology or differentiation between tumors. However, the 5-aza-dC treatment significantly shortened RMS latency time. Thus, the median RMS latency time of 5-aza-dC treated animals was 50 days and that of untreated animals was 98 days (*P* = 0.0012 for palpable RMS by Gehan-Breslow-Wilcoxon test). Together, these data suggest that changes in DNA methylation controls RMS initiation and progression. Furthermore, the 5-aza-dC treatment resulted in an average number of tumors of 3.5 ± 0.49 (Table 1), while the average tumor number of untreated mice was 2.6 ± 0.29 per animal (*P* = 0.217). Despite a lack of significance these data show that 5-aza-dC, in addition to shortening the latency time, may also increase the multiplicity of RMS.

**Table 1:** Tumor formation in *Ptch*^+/−^ mice after application of 5-aza-dC

age at 5-aza-dC application	n	age range; median (days)	mice with RMS^1^	median LT^2^ of RMS (days)	RMS^3^/mouse (mean ± SEM)	mice with cystic tumors^4^	others
E9.5–E11.5	25	46–230; 101	23 (92%)	50	3.5 ± 0.49	6 (24%)	2^5^
–	21	93–272; 161	20 (95%)	98	2.6 ± 0.29	6 (29%)	0

1 palpable RMS only

2 latency time of the first palpable RMS

3 palpable and non-palpable RMS

4 see [13]

5 1 mouse suffered from rectum prolapse, 1 developed a non-palpable RMS

Since *Ptch*^+/−^ mice are highly susceptible to RMS formation at E9.5, whereas the susceptibility progressively declines at later stages, we next hypothesized that the *Ptch* promoter may show a different methylation pattern at different developmental stages during embryogenesis. To test this hypothesis we compared the methylation status of the *Ptch* locus at the RMS-susceptible stage E9.5 with the RMS-non-susceptible stage E13.5. To this end we analyzed the methylation-sensitive regions 3, 4 and 7 (see Fig. 1) in DNA from 30 E9.5 and 4 E13.5 wt embryos. As shown in Supplemental Fig. S7 almost no DNA methylation of the *Ptch* promoter was detectable in E9.5 embryos. At E13.5 the methylation increased and was significantly higher compared to E9.5 (*P* = 0.0238, *P* = 0.0159, *P* = 0.0333 for amplicons 3, 4 and 7, respectively; for control experiments see supplemental Fig. S8). Together, these data show that the *Ptch* promoter at the RMS-susceptible stage (i.e. at E9.5) is marginally methylated and methylation increases at the RMS-non-susceptible (i.e. at E13.5) stage. Along with the observations that the 5-aza-dC-treatment at the RMS-susceptible stage accelerates RMS growth (i.e. 5-aza-dC accelerated occurrence of palpable RMS) these data support our notion that *Ptch* promoter hypomethylation is involved in RMS formation.

## DISCUSSION

In this study we analyzed epigenetic modifications at the *Ptch* promoter in RMS of heterozygous *Ptch* mice. Since only the mutant *Ptch* transcript is overexpressed in RMS we previously assumed that the tumor-initiating event is active repression of the wt *Ptch* allele [11]. Indeed, our previous data showed that 5-aza-dC-treatment of RMS modulated methylation of *Ptch* upstream sequences. In general, changes in the methylation pattern are a hallmark of cancer cells. Whereas hypomethylated regions in tumor cells usually comprise genes or structures that are normally silent or interfere with chromosomal stability, hypermethylated regions frequently include promoter regions of tumor suppressor genes [27].

In our study the promoter of the putative tumor suppressor gene *Ptch* is hypomethylated in RMS compared to SM in regions with only a moderate CpG content. This fits to findings that DNA methylation is usually associated with CpG-poor DNA, whereas CpG islands remain mostly unmethylated [16]. In addition, the *Ptch* promoter in RMS is furthermore occupied by the activation-associated histone mark H3K4me3. Indeed H3K4me3 is a hallmark of unmethlyated CpG islands [28]. Accordingly, fragment 3 (see Figs. 1, 2) showed both DNA methylation and H3K4me3 occupancy, and the level of H3K4me3 signal correlated inversely with the degree of DNA methylation. These data strongly suggest that the *Ptch* promoter in RMS is activated due to hypomethylation and increased occupancy of activating histone marks. Interestingly accumulation of H3K4me3 has been shown to be associated with strong gene activation in tumor tissues even without changes in DNA methylation levels [29]. This sheds light on the promoter regions without methylation changes in RMS and SM (fragments 1, 2 and 5) with increased H3K4me3 occupancy in RMS and supports our assumption that the mutant *Ptch* promoter is activated in RMS by diverse epigenetic mechanisms. Furthermore H3K4me3 protects DNA from methylation [30]. Thus increased H3K4me3 occupancy of the mutant *Ptch* allele might contribute to the pathological *Ptch* expression in RMS.

Hypomethylation of the *Ptch* promoter in RMS is most likely established independently of the tumor-specific expression profile of epigenetic modifier enzymes, because RMS i) generally express elevated levels of genes involved in the establishment and maintenance of genomic hypermethylation (i.e. HDACs and Dnmts; see Fig. 3) and ii) do not exceedingly express genes associated with the regulation of H3K4 methylation.

So far only a few studies analyzed *Ptch* promoter methylation in cancer. Apparently the methylation status varies significantly at different regions of the *Ptch* promoter. As shown in Fig. 1 the *Ptch* promoter contains several alternative first exons. Lack of methylation at the alternative exon 1B was reported for human medulloblastoma [31, 32] whereas others showed methylation in exon 1A [33]. Methylation of exon 1A was also found in gastric cancer samples [34] and exon 1B was methylated in fibroma and dermoids [35], in basal cell carcinoma [36] but not in squamous cell cervical cancer or ovarian cancer [37]. Moreover it was reported that the *Ptch* promoter in medulloblastoma from *Ptch* heterozygous mice is not methylated [38]. Since our previous studies did not reveal methylation near the Gli-binding site 1 in the murine *Ptch* promoter [11], we focused on the CpG islands B-D (Fig. 1) and neighboring CpG rich regions. Strikingly, the hypomethylated *Ptch* promoter regions in RMS cover one of the three Gli-binding sites. Since loss of Ptch function results in aberrant Hh signaling activity that culminates in activation of Gli transcription factors, hypomethylation of the Gli-binding site could explain the very high *Ptch* transcripts levels in tumors of *Ptch*^+/−^ mice.

**Figure 5:**
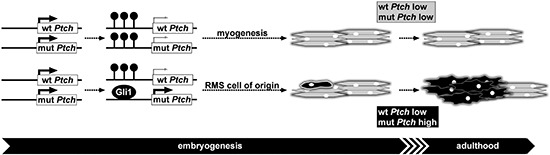
Mechanism of RMS formation in heterozygous *Ptch* mice Initially the *Ptch* promoter is not methylated during early embryonic development allowing abundant expression (thick arrows) of wildtype (wt) and mutant (mut) *Ptch* transcripts in heterozygous *Ptch* knockout mice. Subsequently methylation (resembled by lollipops) of the *Ptch* promoter starts at E13.5 or later during embryogenesis leading to decreased *Ptch* expression of the methylated alleles (as indicated by thin arrows). If both *Ptch* alleles are methylated myogenesis proceeds unperturbed and wt as well as mut *Ptch* are expressed at low levels during and after myogenesis (indicated by grey color). If the mut allele remains accidentally unmethylated and Gli1 binding is increased, the cell of origin for RMS (single black cell) expresses high levels of mut *Ptch* (indicated by black color) and finally gives rise to RMS (cluster of black cells) during adulthood.

These data support a new model of RMS formation in *Ptch*^+/−^ mice (Fig. 5). According to this model the *Ptch* promoter becomes methylated and inactivated after E9.5 in the course of muscle development. This assumption is supported by i) a lack of methylation of the methylation-sensitive sites within the *Ptch* promoter at E9.5, ii) an increase in methylation at E13.5 and iii) distinct methylation of the methylation-sensitive sites in adult SM. It is also fostered by the very low *Ptch* expression in SM [11, 14, 39]. According to our new model a RMS develops when methylation of the mutant *Ptch* locus in a specific RMS precursor cell does not occur during the RMS-susceptible state of embryogenesis (i.e. before E13.5) which then results in increased Gli1 binding and permanent Hh pathway activity.

The finding that 5-aza-dC treatment of E9.5 old *Ptch*^+/−^ embryos accelerates RMS growth also favors this model of RMS formation. 5-aza-dC irreversibly inhibits Dnmts. Whereas Dnmt1 is the primary enzyme responsible for copying methylation patterns after DNA replication, Dnmt3a and Dnmt3b are responsible for *de novo* methylation of genes, also during mammalian development [40, 41]. Indeed, *de novo* DNA methylation in the embryo occurs between E4.5 and E13.5 [16] and Dnmt3a and Dnmt3b are expressed ubiquitously in the E9.5 old embryo [40]. Since the *Ptch* promoter is unmethylated at the RMS-susceptible stage E9.5 (Supplemental Fig. S7) and since 5-aza-dC blocks both the *de novo* and maintenance Dnmts [42], blockage of *de novo* methylation of the *Ptch* promoter at E9.5 (by 5-aza-dC treatment) appears to be responsible for maintaining the mutant *Ptch* locus in an unmethylated state. If this occurs in a RMS precursor cell, the pathological activation of Hh signaling provides a proliferative advantage. In addition to this mechanism, 5-aza-dC likely also affects other DNA regions which may contribute to accelerated RMS formation in *Ptch*^+/−^ mice.

At first glance, our new model appears to conflict with our recent observation that heterozygosity for *Dnmt1* delayed (instead of accelerated) the growth of medulloblastoma in heterozygous *Ptch* mice. Medulloblastoma formation in *Ptch* mutant mice follows a similar molecular mechanism as RMS formation with the mutant *Ptch* transcripts being highly overexpressed and the wt transcripts underrepresented [11]. However, since the reduction of Dnmt1 activity in heterozygous *Ptch* mice did not affect initiation of medulloblastoma [11] and since reduced levels of Dnmt1 activity can inhibit proliferation and induce apoptosis [43], it is possible that the already initiated medulloblastoma simply underwent pronounced apoptosis and thus stagnated in their growth.

Furthermore, we showed that treatment with 5-aza-dC of adult mice did not influence RMS growth [11]. This indicates that 5-aza-dC-induced demethylation of the wt *Ptch* locus in already established RMS precursor lesions in the adult does not compensate for the overexpression of the demethylated and thus overexpressed mutant *Ptch* allele. The efficient prevention of RMS growth by 5-aza-dC along with valproic acid in the adult animal [11] can be explained either by a more efficient reactivation of wt Ptch expression after combination therapy or by additional Ptch-independent mechanisms.

In conclusion, our data suggest an alternative model of tumor formation caused by the tumor suppressor gene *Ptch*. This model includes loss of methylation and occupancy by activating histone marks and probably Gli1 binding of the mutant *Ptch* locus. In this model, overexpression of the mutant, non-functional Ptch protein results in aberrant Hh signaling activity and tumor formation. Whether this model can also be applied to human Ptch-associated tumors remains to be analyzed in the future.

## METHODS

### Compounds

5-aza-dC (Sigma-Aldrich, Taufkirchen, Germany) was diluted in PBS. Stocks of 120 μg/ml 5-aza-dC were stored at −80°C and the required amount was diluted from a new aliquot directly prior use.

### Animals

Experiments using animals were performed with consideration of all necessary legal requirements. *Ptch*^+/−^ mice, which served for treatment studies and tumor monitoring, were on a Balb/cJ background that confers RMS susceptibility [44]. In *Ptch*^+/−^ mice exons 8 and 9 of the murine *Ptch* gene are replaced by a *neo* cassette. Genotyping of the *wt* and the targeted *Ptch* alleles were performed as described [13]. Some of the results were confirmed in *Ptch^neo67/+^* mice that lack *Ptch* exons 6 and 7 (for description of the mice and genotyping see Hahn *et al.* [14]).

Balb x B6 *Ptch^+/^*– mice used for SNP analyses were on a mixed Balb/cJ x C57BL/6 background. In these mice the wt *Ptch* locus is derived from a C57BL/6 wt female whereas the mutated *Ptch* locus is derived from a *Ptch*^+/−^ male on a Balb/cJ background.

### 5-aza-dC injection

To treat embryos *in utero* with 5-aza-dC, wt Balb/cJ females were mated to *Ptch*^+/−^ males. The pregnant females were injected intraperitoneally with 100 μg/kg of 5-aza-dC (maximal tolerable dose; see Ecke *et al.* [11]) in PBS at 9.5, 10.5, and 11.5 dpc. For determination of embryonic ages, noon on the day of the postcoital plug was taken as E0.5.

### Tumor monitoring and histological examination

RMS development in *Ptch*^+/−^ mice was monitored by weekly manual palpations of animals as most RMS develop in the muscles of the hip/thigh or intraabdominally. Tail deformation in some mice treated with 5-aza-dC during embryogenesis indicated that they were efficiently exposed to the substance during embryogenesis [45, 46]. If possible, the observation period encompassed 250 days. After death the animals were examined for additional, non-visible and non-palpable tumors. The identity of the tumors as RMS was confirmed using paraffin sections stained with hematoxylin and eosin.

### DNA and RNA-isolation and qPCR

DNA was extracted according to standard procedures. Total RNA was isolated using PeqGOLD TriFast™ reagent (PeqLab, Erlangen, Germany). Reverse transcription of total RNA was performed using random hexamers and SuperScriptII reverse transcriptase (Invitrogen, Karlsruhe, Germany). qPCR was performed on an ABI PRISM 7900HT Sequence Detection System (Applied Biosystems, Foster City, California, USA). Amplification of 18s rRNA as an endogenous control was performed to standardize the amount of sample RNA. All primers and probes used in qPCR assays for analyses of transcript expression were intron-flanking (supplemental Table 1).

In order to distinguish between the wt *Ptch* and mutant *Ptch* transcripts derived from *Ptch*^+/−^ mice we established a TaqMan assay using fluorogenic probes that span exons 8 and 9 or exons 7 and 10, respectively (supplemental Table 1).

### MeDIP

MeDIP was performed with phenol chloroform extracted DNA from RMS and the corresponding SM from *Ptch*^+/−^ mice and from E9.5 and E13.5 mouse embryos. The MagMeDIP kit (Diagenode, Liège, Belgium) was used according to the manufacturer's instructions. Efficiency of DNA shearing (Bioruptor^®^ NGS, Diagenode, high power, 5 cycles: 30 sec on/off) was analyzed by agarose gel electrophoresis. MeDIP and input samples were analyzed by qPCR and MeDIP samples were normalized to the respective input. The results are shown as % input (ratio signals in immunoprecipitated DNA versus input DNA).

### Bisulfite next generation sequencing

Sodium bisulfite treatment of genomic DNA from RMS and SM from 9 *Ptch*^+/−^ mice was carried out using the EpiTect Bisulfite Kit (Qiagen, Venlo, Netherlands) according to the manufacturer's instructions. Bisulfite-treated DNA was amplified with primers located in the CpG island A (Fig. 1) and in fragments 3–7 specific for bisulfite-treated DNA (see supplemental Table 1). For primer design Methyl Primer Express Software v1.0 (Applied Biosystems) was used. All gel extracted amplicons (QIAquick Gel Extraction Kit, Qiagen) derived from one RMS or SM tissue sample were pooled and quantified using Quanti Flour (Promega, Fitchburg, WI, USA). For each sample 1.2 μg DNA were diluted in 100 μl tris/EDTA buffer and sonicated to 200 bp using the Nano NGS-Bioruptor^®^ (Diagenode). Library preparation was performed using the TruSeq custom Amplicon Library Preparation (Illumina, San Diego, CA, USA). Library sizes were evaluated using the Bioanalyzer 2100 (Agilent Technologies, Santa Clara, CA, USA) and the quantity was estimated using Quanti Flour. Libraries were diluted to 8 pM and sequenced with the MiSeq System (MS-102–3001, Illumina) using the 300 cycle kit and v3 Reagents.

Bisulfite NGS data were analyzed with the software Sequence Pilot Version 4.1.2 (JSI medical systems, Ettenheim, Germany). Briefly, the sequences (FASTQ files) were aligned to the bisulfite modified reference sequence for each amplicon assuming all C in a CpG position as methylated. The percentage of C to T nucleotide changes compared to the reference sequence was used to calculate the ratio of DNA methylation. The percentage and coverage of C to T nucleotide changes for all CpG in fragments 3, 4 and 7 are shown in supplemental Table 4. The efficiency of bisulfite conversion was estimated by the proportion of non-CpG cytosines converted to uracil and was > 98% in all samples.

### Chromatin immunoprecipitation

The H3K4me3 ChIP was essentially carried out as described [47]. See supplemental Table 2 for buffers and solutions. Briefly, RMS and SM were minced and crosslinked. After washing (PBS) samples were homogenized, dounced, sonicated (Bioruptor^®^ Plus, high power, 30 cycles: 30 sec on/off) and insoluble remains removed by centrifugation. Fifteen μl were used for DNA isolation and quantification (NanoDrop 8000, Thermo Scientific, Waltham, Massachusetts, USA). DNA fragmentation was confirmed by agarose gel electrophoresis. The chromatin was pre-cleared (1 h, 4°C). One μg chromatin was mixed with 1 ml IP buffer/1 μg anti-H3K4me3 or IgG control antibody (see supplemental Table 3) and incubated at 4°C overnight. To bind immunoprecipitated complexes protein A Sepharose was used (2 h, 4°C). Thereafter samples were washed with IP buffer/0.1% SDS, wash buffer, IP buffer/0.1% SDS and TE. Then the immunoprecipitated complexes and input samples (0.1 μg) were incubated with 10% chelex 100 (10 min, 95°C). Two μg proteinase K were added and samples were heated to 55°C (30 min) and 95°C (10 min). Samples were centrifuged and supernatants were used for qPCR. Therefore all input samples were diluted 1:10 with water. ChIP samples were normalized to their respective input sample. The results are shown as % input.

The Gli1 ChIP was conducted using a modification of the Magna ChIP kit protocol (EMD Millipore, Bedford, MA). Defrosted SM and RMS tissue samples were sliced with a scalpel blade, resuspended in PBS and fixed at room temperature with 1% formaldehyde for 15 min. After that, 10x glycine solution was added to quench unreacted formaldehyde, samples were spinned down and washed twice with PBS/proteases inhibitor cocktail (Roche, Mannheim, Germany). Samples were minced for 2 min using the homogenizer, resuspended in cell lysis buffer, kept on ice for 40–45 min and vortexed for 5 min. After spinning down samples were resuspended in nuclear lysis buffer, sonicated for 40–45 cycles and insoluble remains were removed by centrifugation. The following steps were the same as the ones from H3K4me3 ChIP.

### SNP analysis

After Gli1 ChIP and qPCR using primers encompassing the SNP rs29624336 (Supplemental Table 1) the amplicons were purified with the QIAquick Gel Extraction Kit (Qiagen) and cloned into pGEM-T Easy (Promega). For each amplicon at least 40 clones were sequenced using the BigDye Terminator v3.1 Cycle Sequencing Kit (Applied Biosystems) according to the manufacturer's instruction. The sequences were analyzed on the ABI 3500xL Genetic Analyzer (Applied Biosystems) and the chromatograms were evaluated using the software BioEdit version 7.1.3.0 [48].

### Western blot

RMS and SM from 4 heterozygous *Ptch* mice were homogenized in 1% Triton X-100, 150 mM Hepes, 1 mM EDTA at pH 7, supplemented with protease and phosphatase inhibitors. For determination of protein concentration the Pierce Protein BCA Assay Kit (Thermo Scientific) was used. Fifty μg of each sample were transferred using standard methods by semi-dry blotting onto a PVDF membrane (GE Healthcare, Buckinghamshire, England). Antibodies are shown in supplemental Table 3.

### Analysis and visualization of ChIP-sequencing (ChIP-seq) data

Raw FASTQ files for H3K4me3 ChIP-seq (GSM721293) and input (GSM721307) from differentiated C2C12 cells were downloaded from the European Nucleotide Archive and analyzed with a Galaxy server. Reads were mapped to the mouse genome (mm9) using Bowtie (version 1.1.2) and wiggle files were generated using MACS (version 1.0.0). ChIP-seq data for H3K4me3 from mouse embryonic stem cells (GSM1033636) were downloaded as processed wiggle files directly from the NCBI Gene Expression Omnibus. Tracks for transcriptome data from the transcriptome analyzer (TromER) project as well as GC content for the region near the *Ptch1* gene were downloaded directly from the UCSC genome browser for visualization together with ChIP-seq data using the Integrated Genome Viewer (IGV version 2.3.25).

### Statistics

GraphPad Prism was used for statistical analysis. Gehan-Breslow-Wilcoxon tests were performed to assay statistical differences for survival curves. A factor was considered statistically significant if it had a two-tail *P* value of < 0.05. Gene expression and tumor multiplicity were analyzed by Mann-Whitney tests. *P* values of < 0.05 were considered significant.

## SUPPLEMENTARY DATA




