# Supplementary material for: Overexpression of mutant Ptch in rhabdomyosarcomas is associated with promoter hypomethylation and increased Gli1 and H3K4me3 occupancy
Source: Oncotarget. 2015 Mar 14;6(11):9113–24. doi: 10.18632/oncotarget.3272 (PMC4496206; doi:10.18632/oncotarget.3272)
Supplement: Supplementary file 1 [file oncotarget-06-9113-s001.pdf]

SUPPLEMENTARY DATA

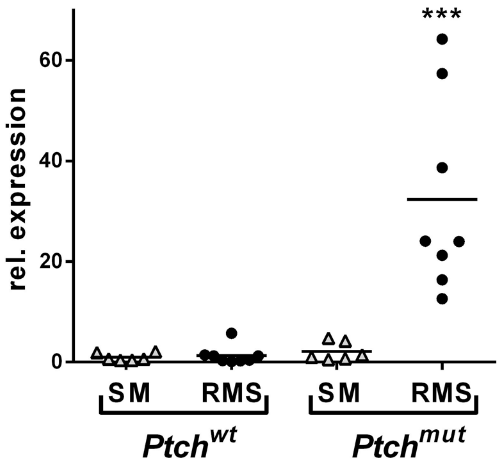

Supplementary Figure S1: Increased expression of mutant *Ptch* transcripts in RMS of heterozygous *Ptch* mice. Probe based qPCR was carried out to determine the expression of wildtype (wt) and mutant (mut) *Ptch* transcripts in 6 SM and 8 RMS samples from $Ptch^{+/-}$ mice. The wt *Ptch* expression in SM was set to 1 and each sample is represented by triangles (SM) and dots (RMS). The mean values are indicated by lines. The differences between wt *Ptch* in SM and RMS as well as mut *Ptch* in SM each compared to mut *Ptch* in RMS are statistically significant (***$P < 0.001$).

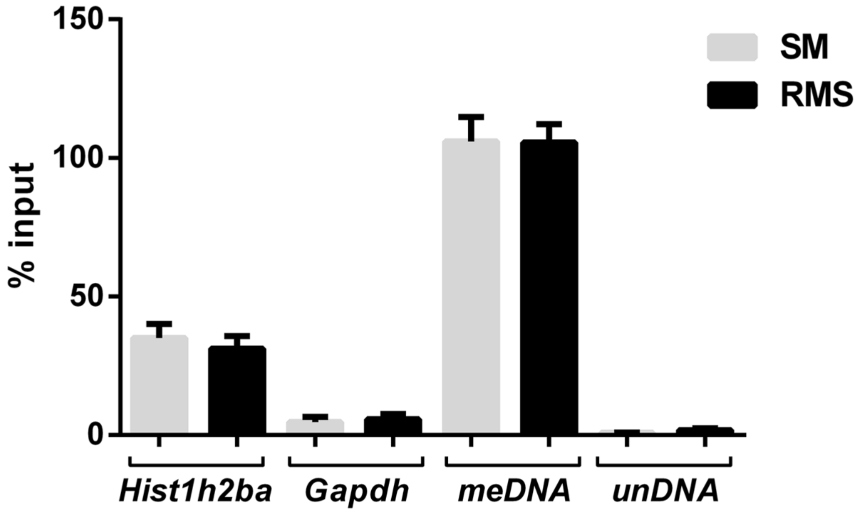

Supplementary Figure S2: Positive and negative controls for MeDIP with RMS and SM DNA. Amplification of the *Hist1h2ba* and *Gapdh* genes served as positive and negative controls for methylated and unmethylated DNA samples, respectively, [1, 2] after MeDIP of DNA derived from RMS and SM. Calculation of % input is described in the material and methods sections. Furthermore, methylated (meDNA) and unmethylated DNA (unDNA) provided by the MagMeDIP kit were added to each sample. After MeDIP the DNA was amplified using oligonucleotides provided by the kit.

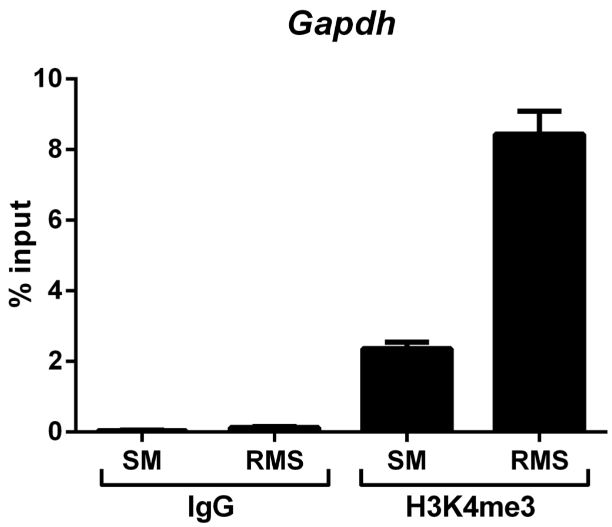

Supplementary Figure S3: Positive control for H3K4me3 enrichment. Amplification of the *Gapdh* gene served as a positive control for ChIP with the anti-H3K4me3 antibody in RMS and SM. Data are shown as % input. As a negative control the samples were immunoprecipitated with IgG instead of anti-H3K4me3 antibody.

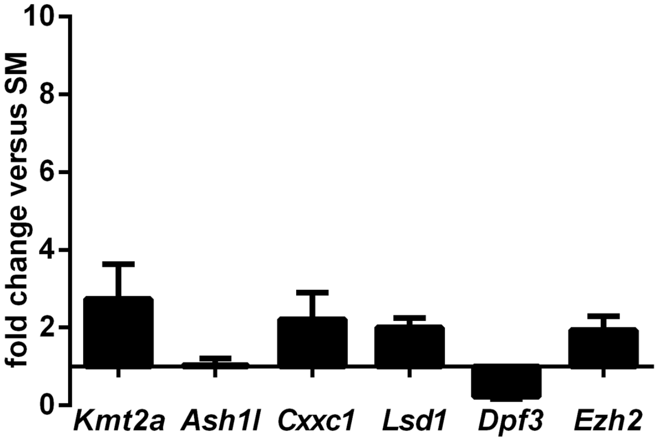

Supplementary Figure S4: Expression profile of epigenetic modulators in RMS and SM. qPCR of *Kmt2a* (*n* = 5), *Ash1l* (*n* = 5), *Cxxc1* (*n* = 5), *Kdm1a (Lsd1) (n = 4)*, *Dpf3* (*n* = 4), and *Ezh2* (*n* = 6) of cDNA derived from RMS and SM of heterozygous *Ptch* mice. Shown is the fold expression (mean ± SEM) in RMS to the respective control SMs that were set to 1. However, the differences between RMS and SM were not statistically significant.

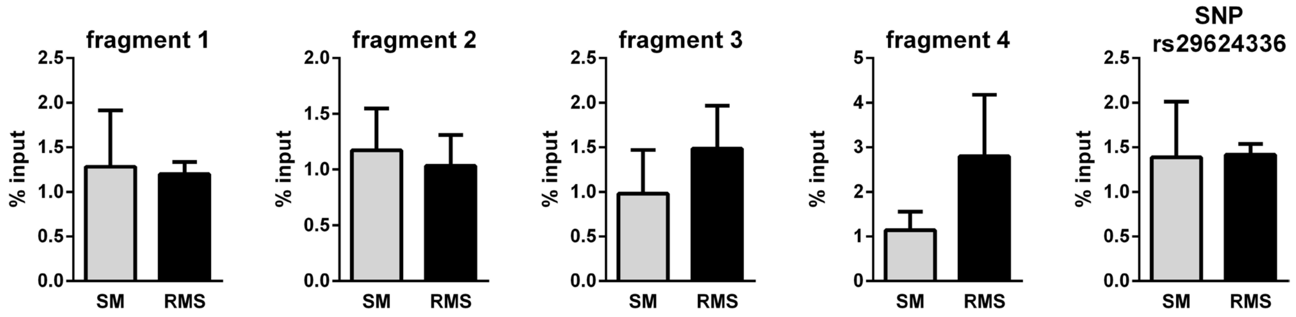

Supplementary Figure S5: Increased Gli1-binding to the Gli-BS 2 in RMS. The ChIP enrichment for Gli1 was analyzed in chromatin from RMS (black bars) and SM (grey bars) derived from 5 *Ptch*$^{+/-}$ mice and quantified by qPCR. Mean values and SEM for the amplicons 1, 2, 3, 4 and the amplicon covering SNP rs29624336 are shown. IgG served as a negative control (data not shown). Amplicon 4 covers the Gli-BS 2 (see Fig. 1).

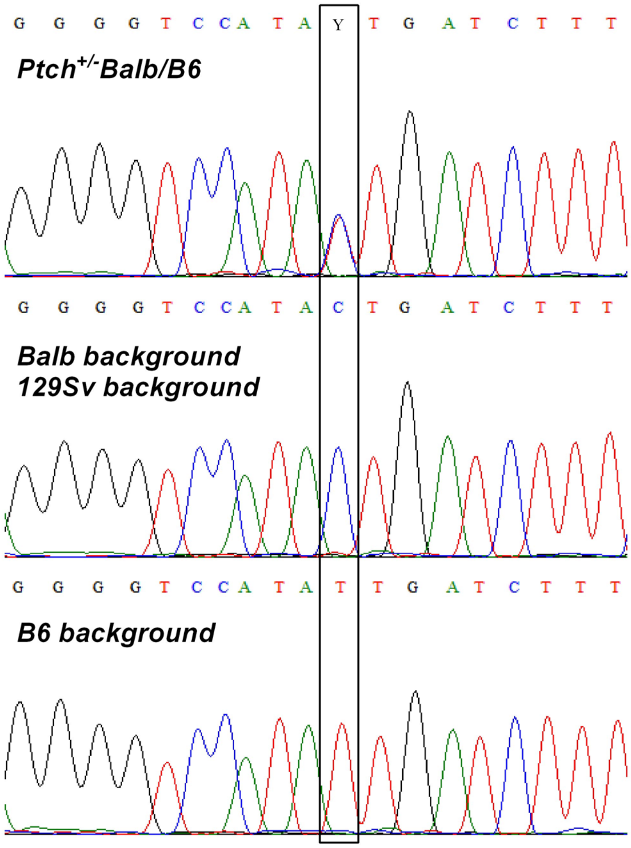

Supplementary Figure S6: Confirmation of the SNP rs29624336 in *Ptch+/–Balb/B6*, Balb/c, 129Sv and C57BL/6 mice. Genomic DNA of each mouse line was sequenced to confirm the SNP rs29624336 for the respective mouse lines (heterozygous C/T in *Ptch+/–Balb/B6*, C in Balb/c and 129 Sv and T in C57BL/6). Representative chromatograms for each variation are shown.

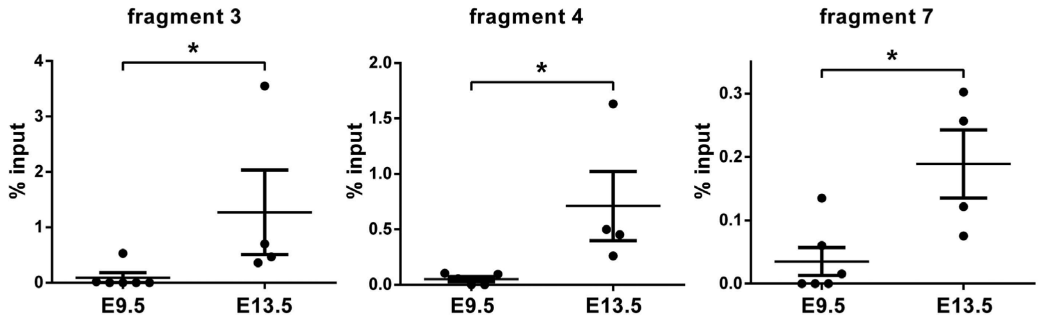

Supplementary Figure S7: Methylation profile of the *Ptch* promoter at E9.5 and E13.5 during embryogenesis. DNA derived from tissue samples from wildtype embryos was subjected to MeDIP and analyzed by qPCR. Each dot represents one sample measured in triplicates. For E9.5 each sample consists of 6 embryos. Mean values (lines) and SEM are indicated for each group. The increased methylation in amplicons 3, 4 and 7 at E13.5 is statistically significant (*$P < 0.05$).

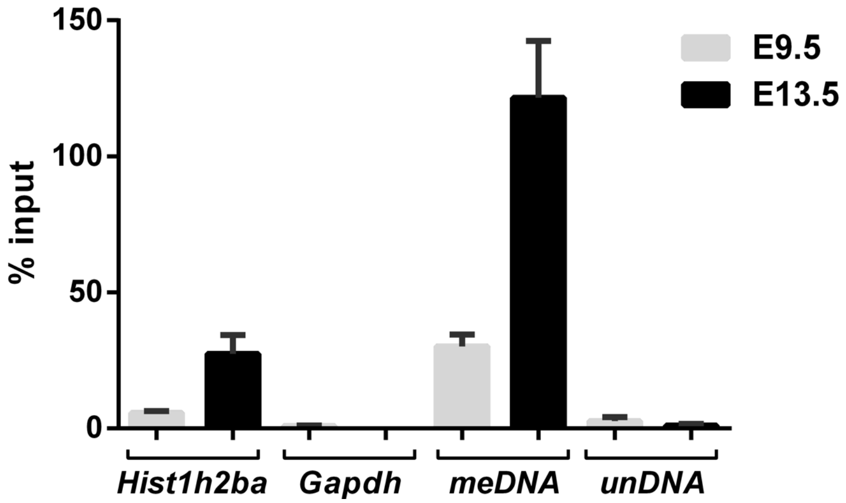

Supplementary Figure S8: Positive and negative controls for MeDIP with E9.5 and E13.5 DNA. Amplification of the *Hist1hba* and *Gapdh* genes served as positive and negative controls for methylated and unmethylated DNA samples, respectively, after MeDIP of DNA derived from E9.5 and E13.5 old mouse embryos. In addition, we also used methylated (meDNA) and unmethylated DNA (unDNA) provided by the MagMeDIP kit as positive and negative controls, respectively. As also shown by the MagMeDIP kit manufacturor's manual the % input of the endogenous positive control (here *Hist1h2ba*) and the meDNA positive control can vary considerably between different samples.

Supplementary Table S1. Oligonucleotides used for qPCR, bisulfite NGS and SNP sequencing

Name	Sequence (5′-3′ orientation)	Location	Application
Ptch-F Wt TQ	TGCAAACCATGTTCCAGTTA	exon 8	*Wt Ptch* expression
Ptch-R1 Wt TQ	TTGGGGCGACACTTTGAT	exon 9	
Ptch Wt Sonde	FAM-ACCACCTCCACGTAAGTCCTCT-BHQ1	exon8/9	
Ptch-F d8/9 TQ	CGTCAAGAATGCCACTGG	exon 7	*Ptch*$^{\Delta 8/9}$ expression
Ptch-R d8/9 TQ	AAACCTGAGTTGTCGCAG	exon 10/11	
Ptch d8/9 Sonde	FAM-AACTTGTCAGCTTGCCTATGCC-BHQ1	exon 7/10	
PtcPrFra3-F_Neu	AGCCATTGGCTGGCAGCTGA	−2601 to	MeDIP Fragment 1
PtcPrFra3-R_Neu	ACGTGACCTGGACCGAGAG	−2304[1]	*Ptch* promoter
PtchProFra4-Af1	GGTTTGGGATTCAGGATGGCAAG	−2827 to	MeDIP Fragment 2
PtcPrFr4-Ar1NEU	GACACGCCGTAGTACATTCCTGA	−2576[1]	*Ptch* promoter
PtchProFra5-If2	AGCAGACCACAGGGGAAG	−3665 to	MeDIP Fragment 3
PtchProFra5-Ir2	GCCCAGCTATGCAAAGCCTCT	−3399[1]	*Ptch* promoter
Frag6-F2	TGCAGTTAGCTGGAAGTTAACA	−3958 to	MeDIP Fragment 4
Frag6-R1	CTTTGGGGATCTAATTGGCCT	−3686[1]	*Ptch* promoter
mPtch-Me-F	CTCGCTTGTAGCTCGCACGC	−7445 to	MeDIP Fragment 5
mPtch-Me-R2	GTTGCGTTCAGCCAGGTGTCT	−7055[1]	*Ptch* promoter
mPtch_Ex1a-F	ATTGATGTGAACCTCACGGTC	−7645 to	MeDIP Fragment 6
mPtch-Me-R	GCGTGCGAGCTACAAGCGAG	−7426[1]	*Ptch* promoter
PtchPr2-F	GAGCTGGGGAAGAGTAAGGT	−8385 to	MeDIP Fragment 7
PtchPr4-R	CCTCTCAGAGTAGCTGCCTT	−8217[1]	*Ptch* promoter
mTsh2b_MeDIP-F	TCTCCTTGCGGCATCTCTTAC	exon 1	*Hist1h2ba* gene as
mTsh2b_MeDIP-R	GGCGGTAAAGGGTGCTACTAT	exon 1	MeDIP positive control
GapDH-ChIP2-F	GGGTTCCTATAAATACGGACTGC	exon 1	*Gapdh* gene as MeDIP positive control ChIP negative control
GapDH-ChIP2-R	CTGGCACTGCACAAGAAG	exon 1	
BiZe Df1	GGGGTTTTGGGTAGGTAGG	25 to	Bisulfite NGS
BiZe Dr1	ATACACCTTAAAAATCTACTCCAAAAC	165[1]	CpG Island A
BiZe Bf1	TTTATTTATTGAGTTAAGGAGTTGTTG	−689 to	Bisulfite NGS
BiZe Cr1	CCCAAATCCCCCCCAAAC	−278[1]	CpG Island A
Fra3-BSP-F2	ATGAAAAAAGTAGATTATAGGGGAA	−3673 to	Bisulfite NGS
Fra3-BSP-R2	CTTTAACCCAACTATACAAAACC	−3394[1]	Frag. 3 *Ptch* promoter
Fra4-BSP-F2	GTAGTTAGTTGGAAGTTAATAAATT	−3957 to	Bisulfite NGS
Fra4-BSP-R1	CTACCCCCAATTTACCTCATTACT	−3784[1]	Frag. 4 *Ptch* promoter
Fra5-BSP-F1	GTYGTTTGGGATTTTATTTGTA	−7332 to	Bisulfite NGS
Fra5-BSP-R1	ACRCATCTCTCCAATCTAAATA	−7034[1]	Frag. 5 *Ptch* promoter

(*Continued*)

Name	Sequence (5′-3′ orientation)	Location	Application
Fra6-BSP-F1	GAGAGATAGGGAGAAGAGAGAGTT	−7713 to	Bisulfite NGS
Fra6-BSP-R3	ACAAATAAAATCCCAAACRAC	−7312[1]	Frag. 6 *Ptch* promoter
Fra7-BSP-F1	AGGTGTTGTTTTTTGGAAGTTT	−8369 to	Bisulfite NGS
Fra7-BSP-R2	TAAATAAACAAACTACCCTCCCA	−8074[1]	Frag. 7 *Ptch* promoter
SNPrs29624336-F	AGTGGGACAATGTAGCGAAG	−5444 to	*Gli1 ChIP and*
SNPrs29624336-R	CCTGAGTCAATCTCTGCAGGT	−5248[1]	*SNP sequencing*
Dnmt1.forw	CACCTAGACGACCCTAACCTG	exon 15	*Dnmt1* expression
Dnmt1.rev	AGGTGGAGTCGTAGATGGACA	exon 16	
Dnmt2.F2	TGCACATGTGGTGGCTGCTAT	exon 2	*Trdmt1* expression
Dnmt2.R	CTAGTTGTCCTTGGATCGGTC	exon 4	
Dnmt3a.forw	CATTGATGAGCGCACAAGGGAGC	exon 11/12	*Dnmt3a* expression[2]
Dnmt3a.rev	GGGTGCTCCAGGGTGACATTGAG	exon 13	
Dnmt3b.F	GGTGATGGCAAGTTTTCTGAGAT	exon 8/9	*Dnmt3b* expression[3]
Dnmt3b.R	ACTCCAGCATGGGCTTCAGCT	exon 10	
Hdac1.F	CTGTTACTACTACGACGGGGA	exon 1/2	*Hdac1* expression
Hdac1.R	TGTGAGGACGGTAGATCTCCA	exon 2/3	
Hdac2.F	GTGTGCTACTACTATGATGGTG	exon 1/2	*Hdac2* expression
Hdac2.R	AGCAGTGGCTTTATGAGGCCT	exon 3	
Hdac3.F	AGTGTCCAGATTCATGATGTCC	exon 14	*Hdac3* expression
Hdac3.R	TCCTTGTCGTTGTCATGGTCG	exon 15	
Hdac4.F2	GTCAACATGAGCAGTTGTCCC	exon 3	*Hdac4* expression[4]
Hdac4.R2	CAAACTCCTGCAGCTTCATCTT	exon 5	
Hdac5.F	AACAGAGCACGCTCATAGCAG	exon 11	*Hdac5* expression[5]
Hdac5.R	CTGCATGACCAGCTGCTGCA	exon 12	
Hdac6.F	GTACTTCCCATCGCCTATGAG	exon 22	*Hdac6* expression[6]
Hdac6.R	GATAGATGCCAAATTGTATCCAC	exon 24	
Hdac7.F2	AACCCAGTCTTCCCCAGCAG	exon 11	*Hdac7* expression[7]
Hdac7.R2	AGGAGCACGTGCCGTTCAGA	exon 13	
Hdac8.F	GACTATGCAGCAGCTATAGGAG	exon 4	*Hdac8* expression[8]
Hdac8.R	TGTAGATCCAAATCCACGTAGAG	exon 5	
Hdac9.F	ATCGAACCCAGTCTGCACCTT	exon 9	*Hdac9* expression[9]
Hdac9.R	TGTCTTCCATGGATTGGTCCC	exon 10	
Hdac10.F	AGAAATATGGGCTGCAGAGGAT	exon 5/6	*Hdac10* expression[10]
Hdac10.R	GGCATTTCCCATCCCAACCTG	exon 7/8	
Hdac11.F	ATGGCATGCTGGTGGAGGCT	exon 3	*Hdac11* expression
Hdac11.R	TCGCTCCACAGCCAGCTTCC	exon 5	
Lsd1F.1	AGATGAGCAGATTGAACATTGGA	exon 10	*Kdm1a* expression[11]
Lsd1.R2	GGAACTCGGCTGTGATATCTCTG	exon 11	

(*Continued*)

Name	Sequence (5′-3′ orientation)	Location	Application
Dpf3.F2	CGCCTCTCAGGAAGACCACGAC	exon 6	*Dpf3* expression[12]
Dpf3.R2	GCGTAGTGGTAGCTGAGTCCTG	exon 7	
Ezh2-F2	GAGGTTCAGAAGAGCTGATGA	exon 2	*Ezh2* expression[13]
Ezh2-R2	TAACGGGATGACTTGTGCTGG	exon 4	
mCxx1-F2	GCATGAAGCTGGCAGCCAACC	exon 9/10	*Cxxc1* expression
mCxx1-R2	CGCAGAAGATCTGCAGATCTG	exon 11	
mMll1-F2	ATGACATAAAGGCTGAGCTGG	exon 2	*Kmt2a* expression[14]
mMll1-R	TCAGAGCCACTTCTAGGTCTC	exon 4	
Ash1l-F1	CCAGAGGATACAGAGGCATGA	exon 10	*Ash1l* expression
Ash1l-R1	TGTCCCAGCTGGCATATCCTT	exon 12	

[1]The position refers to the first ATG in *Ptch* exon 1b as +1
[2]*Dnmt3a*: the location of primers is according to ensembl transcript ENSMUST00000020991
[3]*Dnmt3b*: the location of primers is according to ensembl transcript ENSMUST00000109774
[4]*Hdac3*: the location of primers is according to ensembl transcript ENSMUST00000043498
[5]*Hdac5*: the location of primers is according to ensembl transcript ENSMUST00000107152
[6]*Hdac6*: the location of primers is according to ensembl transcript ENSMUST00000115642
[7]*Hdac7*: the location of primers is according to ensembl transcript ENSMUST00000116408
[8]*Hdac8*: the location of primers is according to ensembl transcript ENSMUST00000113616
[9]*Hdac9*: the location of primers is according to ensembl transcript ENSMUST00000073838
[10]*Hdac10*: the location of primers is according to ensembl transcript ENSMUST00000109347
[11]*Kdm1a*: the location of primers is according to ensembl transcript ENSMUST00000116273
[12]*Dpf3*: the location of primers is according to ensembl transcript ENSMUST00000178756
[13]*Ezh2*: the location of primers is according to ensembl transcript ENSMUST 00000081721
[14]*Kmt2a*: the location of primers is according to ensembl transcript ENSMUST00000128768

Supplementary Table S2. Buffers and solutions for ChIP

Buffer/solution & application	Ingredients
Crosslinking solution for crosslinking and subsequent quenching	0.5% formaldehyde/PBS 156 mM glycine (added for quenching)
Homogenization buffer	150 mM NaCl<br>20 mM EDTA (pH 8)<br>50 mM Tris (pH 7.5)<br>0.5% NP-40<br>1% Triton X-100<br>20 mM NaF<br>Complete Protease Inhibitor (Roche, Basel, Schweiz)
Douncing buffer (samples dounced with pestle A)	50 mM Tris (pH 8)<br>10 mM EDTA (pH 8)<br>1% SDS<br>Complete Protease Inhibitor (Roche)
Preclearing buffer	50% Sepharose (w/v) (GE Healthcare) in IP buffer (see below)
IP buffer	150 mM NaCl<br>20 mM EDTA (pH 8)<br>50 mM Tris (pH 8)<br>1% NP-40<br>20 mM NaF<br>0.5% SDS<br>Complete Protease Inhibitor (Roche)
Protein A buffer	50% (w/v) Protein A Sepharose (GE Healthcare) in IP buffer
Wash buffer	100 mM Tris (pH 8.5)<br>500 mM LiCl<br>1% NP-40<br>1% SDS<br>20 mM EDTA (pH 8)<br>20 mM NaF
TE	10 mM Tris (pH 8)<br>1 mM EDTA (pH 8)

Supplementary Table S3. Antibodies used for ChIP and Western blot

antibodies for ChIP	***concentration***
pAb rabbit anti Histone H3 (tri methyl K4) ab8580 Abcam (Cambridge, UK)	1 μg/ml
pAb rabbit control IgG ab46540 and ab 37415 Abcam	1 μg/ml
pAb rabbit anti Gli1 NB600-600 Novus Biologicals (USA)	1 μg/ml
primary antibodies for Western Blot	***dilution***
pAb rabbit anti Hdac1 #2062 Cell Signaling Technology (Cambridge, UK)	1:1000
mAb mouse anti Hspa8 (HSC70) sc-7298 Santa Cruz (Dallas, Texas, USA	1:10000
secondary antibodies for Western Blot	
pAb sheep anti mouse/HRP[1] NA931 GE Healthcare	1:10000
pAb goat anti rabbit/HRP[1] 111-035-045 Jackson Immunoresearch (USA)	1:5000

[1]signals were visualized using the ECL plus detection system (GE Healthcare).
Abbreviations: HRP, horseradish peroxidase; mAb, monoclonal antibody; pAb, polyclonal antibody

REFERENCES

1. Singal R, vanWert J, Bashambu M, Wolfe SA, Wilkerson DC, Grimes SR. Testis-specific histone H1t gene is hypermethylated in nongerminal cells in the mouse. Biology of reproduction. 2000; 63:1237–1244.

2. Liu Y, Quang P, Braggio E, Ngo H, Badalian-Very G, Flores L, Zhang Y, Sacco A, Maiso P, Azab AK, Azab F, Carrasco R, Rollins BJ, et al. Novel tumor suppressor function of glucocorticoid-induced TNF receptor GITR in multiple myeloma. PloS one. 2013; 8:e66982.
